# Pulmonary Talcosis in an Immunocompromised Patient

**DOI:** 10.1155/2016/4678637

**Published:** 2016-06-30

**Authors:** Thanh-Phuong Nguyen, Sowmya Nanjappa, Manjunath Muddaraju, John N. Greene

**Affiliations:** ^1^H. Lee Moffitt Cancer Center & Research Institute, 12902 Magnolia Drive, Tampa, FL 33612-9416, USA; ^2^Department of Internal Hospital Medicine, H. Lee Moffitt Cancer Center & Research Institute, 12902 Magnolia Drive, Tampa, FL 33612-9416, USA; ^3^Critical Care Medicine, SSM St. Mary's Health Center, Saint Louis, MO 63117, USA; ^4^H. Lee Moffitt Cancer Center & Research Institute, 12902 Magnolia Drive, FOB-3, Tampa, FL 33612-9497, USA

## Abstract

The first case of pulmonary talcosis or talc pneumoconiosis related to inhalation of talc during its extraction and processing in mines was described by Thorel in 1896. Pulmonary talcosis is most commonly seen secondary to occupational exposure or intravenous (IV) drug abuse and, occasionally, in excessive use of cosmetic talc. Based on literature review, there has been an increase in reported incidents of pulmonary talcosis due to various forms of exposure to the mineral. We report an 82-year-old man who is diagnosed with Philadelphia chromosome positive pre-B cell acute lymphoblastic leukemia (ALL) treated with palliative imatinib who presented with chronic hemoptysis and dyspnea shortly after his diagnosis. His symptoms were initially thought to be due to an infectious etiology due to his malignancy, immunocompromised state, and radiographic findings until high-resolution computerized tomographic (HRCT) findings showed a diffuse pulmonary fibrosis picture that prompted further questioning and a more thorough history inquiry on his exposure to causative agents of interstitial lung disease. Very often, patients do not recognize their exposure, especially in those whose exposure is unrelated to their occupation. Our case emphasizes the need for thorough and careful history taking of occupational and nonoccupational exposure to known causative agents of interstitial lung disease.

## 1. Introduction

Cosmetic talc is widely used as a dusting and, often, scented powder by many and is generally not considered to be a hazard when used as intended. After inhalational exposure, some talc particles are cleared by the tracheobronchial tree. However, if cleared insufficiently due to prolonged exposure or exposure to high quantities or both, pulmonary talcosis may develop. Talc has been shown to be fibrogenic due to an immunological response [1]. Inhalational talc has been associated with histological patterns of diffuse interstitial fibrosis, nodular fibrosis, and foreign body granulomatosis [2]. It is diagnosed with radiological and histological findings when a consistent history of exposure is present.

## 2. Case Presentation

An 82-year-old man recently diagnosed with Philadelphia chromosome positive pre-B cell acute lymphoblastic leukemia (ALL) treated with palliative imatinib, hydroxyurea, allopurinol, and dexamethasone with chronic pancytopenia presented to the emergency room with complaints of worsening of his hemoptysis and dyspnea. He has a past medical history of coronary artery disease status after angioplasty 15 years ago, chronic obstructive pulmonary disease, nephrolithiasis, and hearing loss due to acoustic trauma.

**Figure 1:**
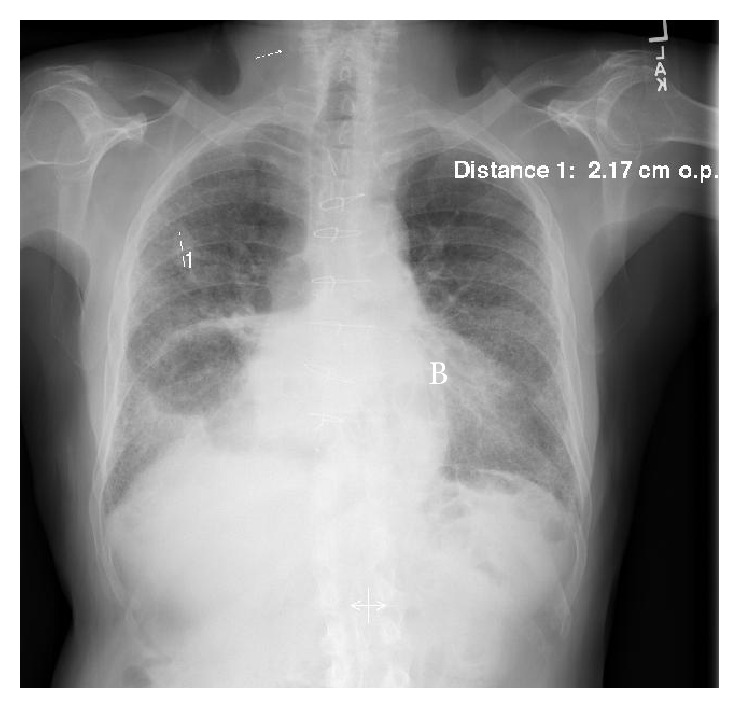
AP chest radiograph showing extensive bilateral reticular-fibrotic pattern with honeycombing and a 2.17 cm nodule in right upper lobe of his lungs.

A few weeks earlier, he began to notice dyspnea on exertion and cough with rust-colored sputum. He had no systemic symptoms, except some mild fatigue and diminished appetite related to his ALL that was improved on dexamethasone. At that time, he had outpatient chest X-rays showing extensive bilateral reticular-fibrotic pattern with honeycombing and mass-like opacity in right lower lobe and a 2.1 cm nodule in right upper lobe of his lungs (Figure 1). Differentials considered included bacterial versus fungal infiltrates versus ALL. He was placed on empiric oral levofloxacin 500 mg daily, which he had only taken for two days. Due to worsening of his hemoptysis and dyspnea, he was prompted to present to the emergency room.

He is a retired sales manager with a smoking history of 30 pack years and quit over 30 years ago and occasionally drinks alcohol but denies illicit drug use.

Significant vitals were as follows: temperature was 97.2°F, heart rate was 90 beats per min (BPM), blood pressure was 138/80 mmHg, respiratory rate was 24–30 breaths per min, oxygen saturation was 90% on room air, and lung sounds were coarse bilaterally with diffuse crackles and decreased breath sounds in right lower lung field. No egophony was appreciated. White blood cell count was 2.91 × 10^9^/L; absolute neutrophil count was 2,450/mm^3^. Hemoglobin was of 8.3 g/dL with platelets of 32,000/mm^3^. Liver function tests were within normal limits except for elevated lactate dehydrogenase of 766 IU/L. Electrolytes are within normal limits. Blood and urine cultures were negative.

**Figure 2:**
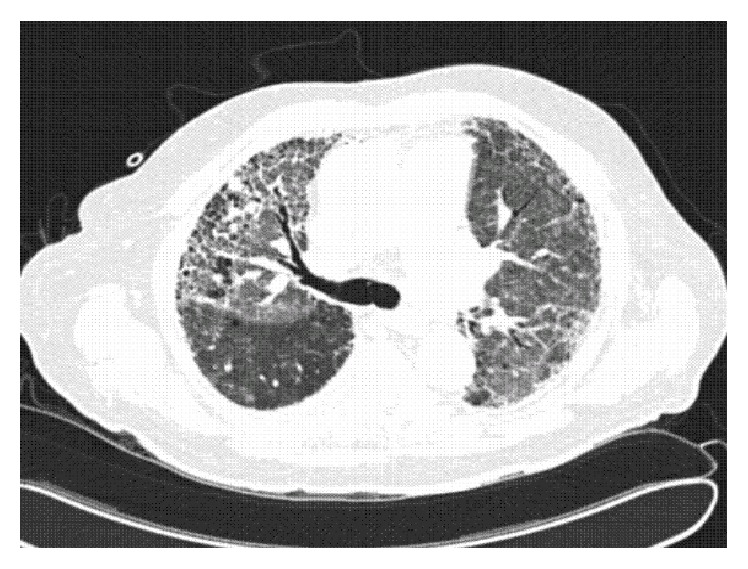
CT angiograph showing no evidence of acute pulmonary thromboembolism but the patient has extensive pulmonary fibrosis.

**Figure 3:**
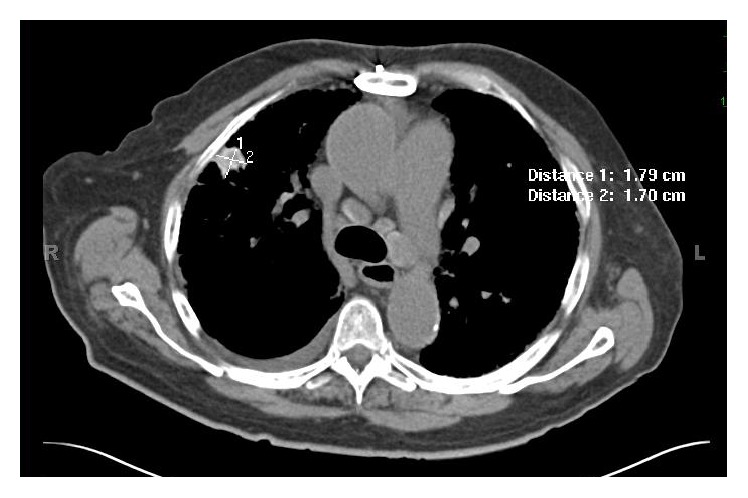
HRCT showing no interval change, when compared to CT angiogram, of multiple right upper lobe pulmonary parenchymal nodules, right pleural effusion, and diffuse fibrotic changes. The most prominent nodule measures 1.8 × 1.7 cm.

CT angiogram revealed no evidence of pulmonary embolism. However, both CT angiogram and subsequent HRCT of the thorax showed evidence of extensive bilateral pulmonary fibrosis and mediastinal lymphadenopathy associated with right pleural effusion, with notable right upper lobe lesion (Figures 2 and 3). The findings prompted a more thorough history taking on exposure to causative agents of interstitial lung disease. He reported that, in his remote past, he would often inhale lavender-scented talcum powder for relaxation during his business trips for an unspecified number of years. He reported history of previous chest X-rays showing evidence of pulmonary fibrosis and pulmonary function tests showing a 20% decrease in lung capacity.

After 2 units of packed red blood cells and 4 units of platelet transfusion, a fiber optic bronchoscopy with bronchoalveolar lavage (BAL) revealed severe interstitial pulmonary fibrosis with severe tracheobronchitis and friability with easy bleeding in the setting of a low platelet count. Cytology of BAL was negative for malignancy. Cultures for fungus, acid-fast bacilli, legionella, nocardia, and pneumocystis jiroveci pneumonia (PJP) and respiratory viruses were all negative. On hospital day 3, he was discharged home on 3 liters of O_2_, levofloxacin 750 mg every other day for 5 days, and fluticasone/salmeterol, in addition to his home medications, with the diagnosis of interstitial lung disease/pulmonary talcosis, tracheobronchitis, thrombocytopenia, and anemia.

He returned to the emergency room one week later with worsening pulmonary symptoms similar to his last admission. Vitals were temperature 36.5°F, heart rate 112 BPM, blood pressure 88/51 mmHg, respiratory rate 32 breaths per min, O_2_ saturation 80% on room air, and 92% on 3 liters of O_2_ via nasal cannula with decreased breath sounds bilaterally without rales, wheezes, or rhonchi. White blood cell count was 1.52 × 10^9^/L with 1+ toxic granulation, hemoglobin 10.0 g/dL, platelet 55,000/mm^3^, erythrocyte sedimentation rate 40 mm/hr, and ferritin 762 ng/mL. Electrolytes are within normal limits. Respiratory viral panel, cytomegalovirus (CMV), and multidrug Methicillin-resistant* Staphylococcus aureus* (MRSA) by PCR were negative. Due to advanced disease progression and terminal illness, a transbronchial or open lung biopsy, which would provide definitive diagnosis, was not pursued.

Pulmonary and infectious disease team was consulted for optimization of his regimen and for evaluation of pneumonia and recommended continuing empiric antibiotics: levofloxacin for upper respiratory infection and voriconazole for fungal coverage. Trimethoprim-sulfamethoxazole and steroids were added for PJP coverage. An echocardiograph for evaluation of pulmonary hypertension revealed severely dilated right ventricle and right atrium with reduced systolic function and severe tricuspid regurgitation with right ventricular systolic pressure of 65–75 mmHg, consistent with severe pulmonary hypertension. Left heart was unremarkable.

Due to his terminal illness and deteriorating clinical course on hospital day 4, he was discharged to hospice with comfort care only. He was placed on basal rate of morphine for respiratory distress. He expired comfortably three days later.

## 3. Discussion

Talc or magnesium silicate [Mg_3_Si_4_O_10_(OH)_2_] is a commonly and widely used agent in industry and daily life. Industrial talc can be found in paints, insecticides, roofing products, asphalt, ceramics, rubber, metal foundries, mining, and even leather. Depending on its use, talc may contain other minerals such as aluminum, iron, calcium, asbestos, and silica [3]. High-purity talc is commonly used as a bulking agent and lubricant in oral medications. Talc is also commonly used as a sclerosing agent for management of malignant pleural effusion. Cosmetically, talc can be found in antiperspirants or body powder [4].

It has been recognized that talc causes disease by two routes: inhalation and intravenously. Pulmonary talcosis, talcosilicosis, and talcoasbestosis may result from occupational exposures to talc dust containing pure or variable amounts of silica or asbestos, respectively. Inhalation of pure talc found in cosmetics has been reported to cause pulmonary talcosis. However, there is no substantial evidence that cosmetic talc, when used as intended, presents a health hazard. Pulmonary talcosis has also been reported in a wide variety of oral medications that are misused intravenously. These include methylphenidate, methadone, promethazine, diazepam, acetaminophen, and many others [4, 5]. Talc particles cause disease by being entrapped in the pulmonary parenchyma or vasculature causing a granulomatous reaction resulting in fibrotic lesions [2].

In a review of several case reports of symptomatic pulmonary talcosis, patients typically presented with initial dyspnea to progressive exertional dyspnea, fatigue, and cough with or without systemic symptoms such as fever, chills, or night sweats [6, 7]. Such was the case for our patient who presented with similar, nonspecific symptoms. While these symptoms may be seen in restrictive pulmonary diseases, such presentation may also be seen in infectious etiologies such as tuberculosis and fungal, bacterial, or viral infections of the lungs. Therefore, it is important to rule out infectious causes, especially in our patient, given his immunocompromised state of chronic pancytopenia in the setting of a hematological malignancy on chronic steroids. It has also been reported that spontaneous pneumothorax was the presenting symptom in a few cases [2, 8]. In a case reported by Caceres et al., a 41-year-old male who presented with complaints of productive cough, dyspnea, fever, and chills was found to have spontaneous pneumothorax and cavitary pulmonary lesions on HRCT. Similar to our case, an extensive workup for an infectious etiology and vasculitis was done. However, all results were negative. It was not until the patient underwent thoracoscopic drainage and pulmonary biopsy, which revealed noncaseating granulomas with lung parenchyma containing birefringent particles, suggesting talc, that the patient was admitted to intravenous use of crushed methadone [8].

When diagnosing pulmonary talcosis, physical exam and laboratory exams are usually unremarkable. There may be some bibasilar crackles [2]. An important clue is a history of occupational exposure or IV drug abuse. However, not all patients will present with such a conspicuous exposure history. Therefore, in such cases, a thorough history, review of systems, and workup of other etiologies are warranted. Chest X-rays are an essential part of the workup. Radiographically, pulmonary talcosis is characterized by generalized haziness, small nodules, and reticulations, either diffuse or predominantly in the lower zones [9]. With disease progression, nodule confluence results in large opacities that resemble those found in progressive massive fibrosis [10]. In some patients, hilar lymphadenopathy develops. A HRCT may be obtained. Typical findings of small centrilobular, subpleural nodules, and heterogeneous conglomerate masses containing high-density amorphous areas, with or without panlobular emphysema in the lower lobes, are highly suggestive of pulmonary talcosis [6, 7]. Moreover, emphysema, if present, is typically centrilobular or apical in inhalational pulmonary talcosis and is typically basal in the intravenous form [2].

Although radiological findings are suggestive, transbronchial or open lung biopsy should be performed. Biopsy is necessary as definitive diagnosis is made by light microscopy of lung tissue specimen. Histologically, granulomas can be visualized as birefringent needle-shaped talc crystals in multinucleated giant cells under polarized light. Two patterns of inflammatory reactions have been described, either fibrosis or granulomatous formation. Histopathology can be diffusely interstitial fibrotic reaction or irregularly nodular or as a noncaseating granulomatous reaction. Nonetheless, if a biopsy is not possible, as in our case due to thrombocytopenia, a bronchoscopy or fine-needle aspiration of pulmonary masses, coupled with an appropriate history, may provide sufficient evidence for the diagnosis [2]. Mineralogical analysis of BAL or pulmonary tissue is an alternative to confirming exposure to talc. Although findings of talc particles are not sufficient for diagnosis, they are helpful in making the diagnosis in the presence of consistent clinical, radiological, or histological data [11, 12]. The presence of these findings in patients with an associated exposure history either by occupational, cosmetological use or by intravenous drug use is highly suggestive.

The natural history of pulmonary talcosis is said to be slowly progressive, even after exposure of dust has ceased [3]. Our case suggests this with a history of a chronic and progressive course of dyspnea and interstitial lung disease long after the cessation of exposure. Development of symptomatic interstitial lung disease many years after a relatively short exposure has been described. Reported cases of this includes symptomatic pulmonary talcosis diagnosed ten years after cessation of a brief four-month ritual of inhaling cosmetic talcum powder and another case of pulmonary talcosis diagnosed more than forty years after a five-year occupational exposure to talc [13, 14]. In another reported case in 1977, a ten-year-old child, who had accidentally inhaled a large quantity of baby talcum powder once at the age of two, was diagnosed with pulmonary fibrosis with evidence of pulmonary hypertension and biopsy evidence of pulmonary talcosis [15]. Long-term complications may include chronic respiratory failure, pulmonary hypertension, and right heart failure or cor pulmonale, as was also seen in our patient's echocardiogram findings. There is no established treatment for pulmonary talcosis with mixed results when steroids and immunosuppressants are used. Transplantation is an option reserved for patients with end-stage disease. Patients are advised to stop exposure and tobacco use [2]. However, fibrotic changes are irreversible and disease may still continue to progress.

In immunocompromised patients with significant comorbidities, such as our patient, the initial presentation may suggest an infectious etiology, necessitating an infectious workup. However, given the radiographic findings of pulmonary fibrosis, other causes of interstitial lung diseases must be considered, in addition to idiopathic pulmonary fibrosis. Therefore, our case serves to emphasize the need for thorough and careful history taking of occupational and nonoccupational exposure to known causative agents of interstitial lung disease.
